# Angioplasty of Dysfunctional Dialysis Fistula or Graft with Resveratrol-Excipient and Paclitaxel-Coated Balloon Improves Primary Patency Rates Compared to Plain Angioplasty Alone

**DOI:** 10.3390/jcm11247405

**Published:** 2022-12-14

**Authors:** Matej Novak, Patrik Matras, Jan Kavan, Lukas Lambert, Andrea Burgetova

**Affiliations:** 1 Department of Radiology, First Faculty of Medicine, Charles University, 121 08 Prague, Czech Republic; 2 Faculty of Health Studies, Technical University Liberec, 461 17 Liberec, Czech Republic

**Keywords:** vascular access, hemodialysis, angioplasty, stenosis, drug-coated balloon, paclitaxel, resveratrol

## Abstract

In this prospective randomized single-blinded study (reg. ISRCTN11414306), 76 patients with a dysfunctional dialysis fistula or graft due to a single de novo or recurrent stenosis in the access circuit were randomized to receive either conventional PTA (POBA) as a standard of care (*n* = 38) or PTA + adjunctive PTA with a drug-coated (paclitaxel–resveratrol matrix) SeQuent^®^ Please OTW balloon (*n* = 38, DCB). Patients were scheduled for follow-up PTA at 3, 6, 9, and 12 months. The time of clinically driven target-lesion reintervention rate (primary patency rate) after the index procedure was analyzed using the log-rank test. The primary patency rates at 12 months after the index procedure were 17% (DCB) vs. 11% (POBA). At 3 months, they were 87% vs. 74%, at 6 months they were 53% vs. 26%, and at 9 months they were 22% vs. 11%. The hazard ratio for DCB was 0.55 (95%CI 0.32 to 0.95). The median time needed for target-lesion reintervention was longer in the DCB group (181 days) than in the conventional PTA group (98 days, *p* = 0.019). We conclude that PTA with the paclitaxel–resveratrol drug-coated SeQuent^®^ Please OTW balloon in patients with de novo or recurrent stenosis in dialysis arteriovenous fistulas or grafts prolongs the time needed for target lesion reintervention and improves primary patency rates in the first year after the index procedure.

## 1. Introduction

Both arteriovenous fistulas (AVFs) and arteriovenous grafts (AVGs) are considered appropriate first-line vascular access routes for hemodialysis patients [1,2]. Both suffer from low primary patency rates. Turbulent flow through the anastomosis site of the vascular access and repeated cannulation lead to neointimal hyperplasia, stenosis, thrombosis, and finally a loss of function and abandonment.

Repeated secondary interventions, mostly percutaneous transluminal angioplasty (PTA), are successful in restoring flow with assisted patency rates of 71% in the first year [3]. Because the primary patency rates after conventional PTA remain disappointingly low due to early restenosis, attempts have been made to reduce the number of reinterventions required during the lifetime of the access, or to at least prolong the time between them [2]. Drug-coated balloon (DCB) catheters have the advantage of leaving nothing behind; therefore, they can be used even in locations where, due to anatomical and technical reasons, stents and stent grafts cannot—mainly in juxta-anastomotic lesions, cannulation segments, or highly mobile or tortuous segments of outflow veins [4].

Although DCBs with paclitaxel as a mitotic inhibitor of neointimal proliferation have been approved for clinical use, their advantage in failing dialysis access in the first year after the index procedure has been questioned [5,6]. Resveratrol is a grape polyphenol that diminishes inflammatory responses in the vessel wall [7]. Resveratrol-excipient and paclitaxel-coated DCBs are therefore expected to provide additional benefits [8].

The objective of this prospective randomized single-blinded trial was to assess the performance of a paclitaxel- and resveratrol-matrix-coated balloon angioplasty catheter (Sequent^®^ Please OTW) in comparison with conventional PTA as the standard of care for dysfunctional AVFs or AVGs due to de novo stenosis or restenosis in the access circuit in terms of primary patency rates.

## 2. Materials and Methods

This study (reg. ISRCTN11414306) was conducted at a tertiary referral center for patients requiring long-term vascular access, mostly for hemodialysis. The study was approved by the Ethical Committee of the General University Hospital in Prague (reference number: 1440/18 S-IV). All patients were given detailed information about the study before signing the informed consent. This investigator-initiated study received no commercial support.

### 2.1. Trial Design

The trial was conducted as a single-center single-blinded prospective randomized clinical study comparing angioplasty with SeQuent^®^ Please OTW (B. Braun Melsungen AG, Berlin, Germany) drug-coated balloon catheters (DCBs) with conventional PTA (“plain old balloon angioplasty”, POBA) as a standard of care in patients with failing AVFs or AVGs due to de novo or recurrent stenosis within the fistula circuit. Only the radiologist knew which balloon was to be used. The referring physician, the patient, and the radiologist performing the follow-up angiography were blinded. The study was designed to randomize 70 patients to achieve a power of 0.8 at a significance level of 0.05, with two equivalent study groups and a survival rate difference of 0.20.

### 2.2. Trial Population, Inclusion/Exclusion Criteria

**Figure 1:**
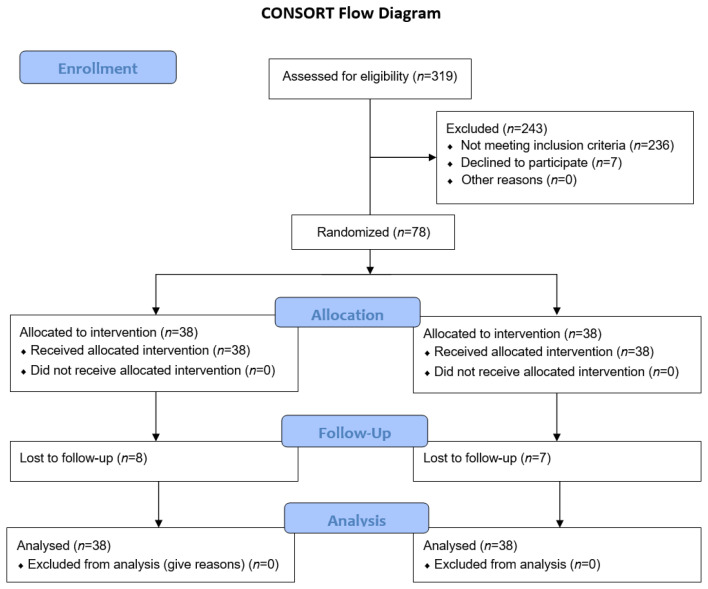
Consort Flow Diagram.

Between October 2018 and October 2020, 76 participants were randomized (Figure 1). All patients were referred to our angiography department, either from a facility that provides them dialysis care or by a consultant vascular surgeon.

The inclusion criteria included the following: (i) age >18 years and life expectancy >1 year; (ii) clinically mature dialysis fistula (AVG or AVF) already used for hemodialysis with an adequate pump speed and dialysis efficacy for at least four consecutive sessions with a two-needle technique; (iii) signs of fistula dysfunction (absent thrill, low flow, high venous pressure, high pulsatility, needling problems, abnormal pulsatility, abnormal auscultation, extremity edema, etc.); and (iv) hemodynamically significant (>50%) stenosis in the juxta-anastomotic or outflow vein of the AVF (excluding central veins defined as veins medial to the lateral margin of the first rib), or in the venous anastomosis, juxta-anastomotic segment, or outflow vein in the AVG (excluding central veins).

The exclusion criteria included the following: (i) access circuit thrombosis in the last year; (ii) history of graft infection; (iii) previous use of a drug-eluting balloon catheter in the access circuit; (iv) in-stent restenosis in a bare-metal or covered stent; (v) contraindications to angiography (e.g., severe contrast media allergy); and (iv) two or more distinct significant stenoses in the access circuit.

### 2.3. Trial Endpoints

The endpoint of the trial was the postinterventional target-lesion primary patency, defined as freedom from clinically driven target-lesion reintervention or thrombotic occlusion of the access circuit during the first 12 months after the index procedure [9]. Clinically driven target-lesion reintervention was characterized as either of the following: (i) angiographic stenosis of more than 50% and signs of fistula dysfunction (absent thrill, low flow, high venous pressure, high pulsatility, needling problems, abnormal pulsatility, abnormal auscultation, extremity edema, etc.); or (ii) stenosis of 70% and more, even without obvious clinical signs of fistula dysfunction.

### 2.4. Trial Device

Patients in the experimental arm of the study received treatment with the SeQuent Please OTW balloon catheter. The balloon was coated with a paclitaxel–resveratrol matrix (3 µg/mm^2^ and 0.9 µg/mm^2^, respectively). Resveratrol (3,5,4′-trihydroxy-trans-stilbene) is a naturally occurring substance commonly found in berries, grapes, and peanuts that acts as an excipient that modulates an appropriate balance between drug adherence to the balloon surface and paclitaxel release [10]. Paclitaxel is a mitotic inhibitor that suppresses neointimal proliferation after an intimal injury during PTA [5].

### 2.5. Trial Procedure

All procedures were carried out in the tertiary vascular access center at the university hospital in the radiology angiography department by one of five board-certified radiologists, or by a senior resident under their direct supervision.

Every procedure started with access-circuit digital subtraction angiography (DSA), with a manual contrast injection through a cannula, which was either inserted in the arterial part of the AVG or directly into the brachial artery in the AVF under manual palpation guidance. The number of acquisitions and projections, as well as the contrast media volume and injection speed, were determined at the discretion of the radiologist.

When significant stenosis was identified, a short 6F sheath was introduced (under local anesthesia) into the access. A 0.035′ guidewire was passed through the stenosis and predilation was performed with a standard or high-pressure balloon catheter with a vessel-to-balloon ratio of 1:1 and a balloon diameter of between 5 and 8 mm. Predilation was deemed successful if there was less than 30% residual stenosis and the absence of rupture or flow-limiting dissection. The balloon was then removed with the guidewire left in place.

**Figure 2:**
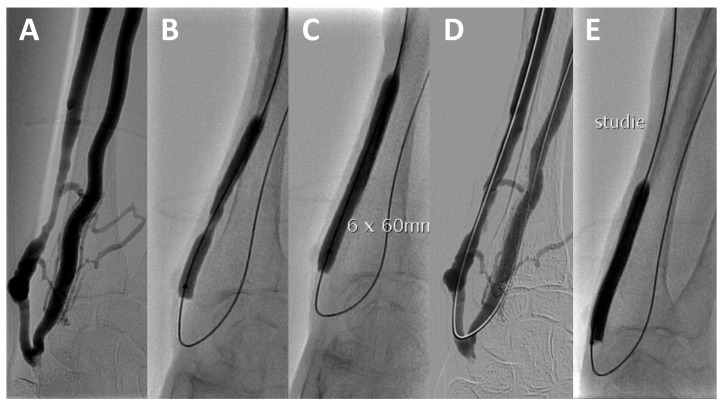
Index PTA in a patient from the DCB group: (**A**) angiography; (**B,C**) conventional PTA; (**D**) after PTA; (**E**) PTA with a drug-coated balloon (DCB) after randomization.

Only after successful predilation were patients assigned to the experimental or control group by permuted block randomization. After that, every patient underwent another angioplasty, either by the same PTA catheter used for the predilation or by the experimental catheter, which involved insufflation to a nominal pressure of the catheter for 3 min (Figure 2). In the DCB group, the balloon length was selected to exceed the target lesion by approximately 10 mm at either end to ensure full coverage and to prevent a geographic mismatch.

### 2.6. Follow-Up

After the index procedure, patients continued their antiplatelet or anticoagulation regimen without change. Follow-up angiographies were scheduled at 3, 6, 9, and 12 months. If the access circuit showed clinical or sonographic signs of dysfunction earlier than the scheduled follow-up, the patient was referred for angiography. DSA of the entire circuit was performed, and a reintervention was either performed or not according to the study protocol.

### 2.7. Cost Analysis

The cost of the initial procedure was calculated as the total reimbursement from the payer, including the cost of the materials and the cost of performing the procedure. In the DCB group, the additional DCB cost was included because it is recommended to perform POBA prior to the use of DCB.

### 2.8. Statistical Analysis

Statistical analyses were performed in MedCalc (MedCalc bvba, Ostend, Belgium) and R (R Core Team, Vienna, Austria). The normality of continuous data was tested using the D’Agostino–Pearson omnibus test. Normally distributed data were compared using the *t*-test. Otherwise, the Mann–Whitney U-test was used. The F-test or χ^2^ test was used to compare the dichotomous variables. Survival curves were plotted using the Kaplan–Meier estimator and compared using the log-rank test. A *p*-value below 0.05 was considered statistically significant.

## 3. Results

Overall, 76 patients were randomized. Baseline patient data, their comorbidities, the type of access, the location of the target lesions, and other data are summarized in Table 1.

The two groups did not show any significant differences in the baseline data.

**Figure 3:**
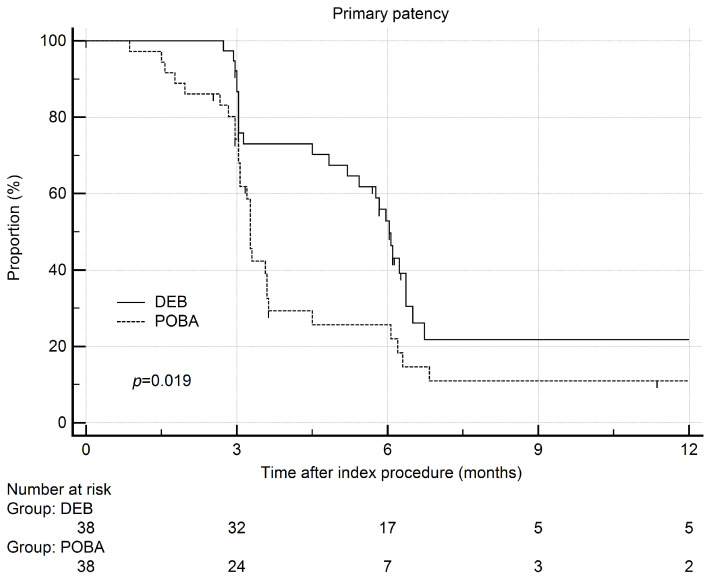
Comparison of primary patency rates after index procedure between patients with conventional angioplasty (POBA) and drug-coated balloon (DCB) on a Kaplan–Meier plot.

The primary patency rates (proportion ± SE) 12 months after the index procedure were 17.4 ± 7.5% (DCB) vs. 11.0 ± 5.9% (POBA). At 3 months, they were 86.7 ± 5.6% vs. 74.2 ± 7.4%, at 6 months they were 52.8 ± 8.4% vs. 25.6 ± 7.9%, and at 9 months they were 21.8 ± 8.1% vs. 11.0 ± 5.9%. The hazard ratio for DCB was 0.55 (95%CI 0.32 to 0.95). The median time needed for target lesion reintervention was longer in the DCB group (median = 181 days, 95%CI 156–91 days) than the POBA group (98 days, 95%CI 92–108 days, *p* = 0.019) (Figure 3). Three (8%) patients in the DCB group and two (5%, *p* = 1.0) patients in the POBA group developed total occlusion (thrombosis). The hazard ratio for DCB was 0.55 (95%CI 0.32 to 0.95). No adverse events were observed.

The total cost of the index procedure in the POBA group was USD 1613 (USD 16.50 per day). The additional cost of the DCB was USD 518. Therefore, in the DCB group, the cost of the initial procedure was increased by 32% (USD 2131 USD, USD 11.80/day).

## 4. Discussion

This study shows that treatment of failing AVFs or AVGs with resveratrol-excipient and paclitaxel DCB improves primary patency rates compared to conventional angioplasty alone. The use of this DCB prolongs the time needed for reintervention.

The deteriorating function of vascular accesses over time and, ultimately, their failures contribute significantly to the morbidity of patients and healthcare system costs [11]. Primary AVF patency rates are low at 51% in the first year. Secondary interventions result in assisted patency (time needed for intervention to maintain or reestablish patency, or to access thrombosis) rates of 78% [12,13]. AVGs have 1-year primary patency rates of 47% and assisted patency rates of 67% [13].

Although the assisted patency rates of AVFs and AVGs are comparable, AVGs require more interventions to maintain usability for hemodialysis [13]. Fistulas tend to develop stenosis most commonly at the juxta-anastomotic site and the outflow vein, while grafts are more likely to develop at the venous anastomosis and the juxta-anastomotic vein [14]. These lesions also represent the majority of the lesions in our study.

Balloon catheter angioplasty, or high-pressure angioplasty, remains the gold standard intervention for a failing fistula when normal-pressure PTA is unsuccessful [1]. However, the primary patency of lesions treated with angioplasty remains disappointingly low at 20–40% in the first year [15,16].

Attempts have been made to prolong the time needed for subsequent intervention using technology that has proven effective in treating peripheral artery disease. Although bare-metal stents show some promise, they have been outperformed by covered stents and new guidelines do not recommend their use [1,17]. Covered stents show very good results in anastomotic stenoses in AVGs and in stent restenosis; however, their use in juxta-anastomotic and cannulation segments (which in AVF are segments where stenoses develop the most) is limited due to anatomical and practical reasons. DCBs with paclitaxel have the advantage of leaving nothing behind.

Paclitaxel is a plant-derived taxine alkaloid. It stabilizes microtubule polymers, prevents their disassembly, and therefore blocks mitosis and inhibits smooth-muscle-cell proliferation, limiting intimal hyperplasia. The highly lipophilic nature of paclitaxel yields passive absorption through the cell membranes and a prolonged effect inside the vessel wall [18]. To facilitate its delivery to the vessel wall, a hydrophilic excipient must be used. Nowadays, over ten different drug-eluting angioplasty catheters are available, and each has its own excipient formula. There are also several types of paclitaxel (crystalline and amorphous), and DBCs can have a mixture of them. Paclitaxel doses range from 2 to 3.5 µg·mm^−2^ of the balloon surface [19]. All these variables lead to the fact that we cannot reliably extrapolate results from one DCB to another and treat them as one group, but evidence must be gathered for each catheter system separately.

The superiority of DCBs in the treatment of peripheral arterial disease compared to POBA has been confirmed in five pivotal randomized controlled trials (RCTs) and drug-eluting technology has almost become a standard of care [20]. The benefit of DCBs in AVFs and AVGs remains controversial. One of the first RCTs by Katsanos et al., published in 2012, showed improved patency rates after DCB angioplasty compared to POBA in failing AVFs and AVGs after 6 months [21]. On the other hand, the first large-scale RCT by Trerotola et al., published in 2018, failed to show a benefit of DCBs at a pre-specified time point of 180 days [22].

An umbrella review by Lazarides et al., published in 2021, showed only a modest benefit of DCBs [23]. A meta-analysis by Chen et al. analyzing more than a thousand patients demonstrated better primary patency rates with DCBs [24]. However, a meta-analysis by Luo et al. in 2022 failed to confirm the benefit of DCBs [5]. What all published studies agree on is the safety of DCBs in the hemodialysis population with no signal of short- or mid-term safety concerns [24,25,26].

In our study, patients treated with DCBs had better primary patency rates compared to patients treated with POBA. The median time needed for lesion reintervention in the DCB group was 181 days compared to the POBA group which was 98 days. The majority of lesions in our study were located in the juxta-anastomotic region and in the venous anastomosis, where the role of intimal hyperplasia is most pronounced. To reduce procedural differences between the groups, randomization was performed only after successful predilation. In the control group, a second angioplasty was also performed with the same balloon used for the predilation after its withdrawal and reinsertion over the guidewire.

Although the use of a DCB was associated with the additional cost of the device, the difference in the median time to the endpoint between the POBA and the DCB groups resulted in lower costs in the maintenance of the patency per time unit in the DCB group. This difference may be higher in countries where the reimbursement for performing the procedure is higher relative to the cost of the material.

Primary patency rates in the POBA group were lower than reported in the literature but are consistent with previous studies by our group [15,16,17]. Our center serves as a tertiary center for vascular accesses and there is a higher concentration of patients with accesses that have a complicated course. In addition, in the context of a prospective study, the vascular access is more consistently monitored for signs of dysfunction and interventions are performed early, which may artificially reduce the primary patency rate (but to the same extent in both groups).

This study has several limitations. First, it is a single-center study conducted in a tertiary care center with a selected population with a possible bias toward more complicated patients. On the other hand, the close collaboration of all interventional radiologists and their expertise in the treatment of vascular accesses ensured that all procedures were performed in a similar manner, which cannot always be guaranteed in multicenter studies. Second, the study is single-blinded because the appearance of DCBs and plain POBA balloon is different. Third, the study groups consisted of both AVFs and AVGs with lesions in different parts of the circuit, making the study sample heterogeneous. Fourth, due to predilation of the stenotic segment with a plain PTA balloon, the drug delivery to the vessel may not have been as efficient as it would have been with primary PTA with DCBs. Finally, this study did not collect any quantitative data on the clinical performance of vascular access.

## 5. Conclusions

This study showed that the treatment of failing AVFs or AVGs with a resveratrol-excipient and paclitaxel-coated balloon improves primary patency rates compared to conventional angioplasty alone. The use of this DCB prolongs the time needed for reintervention.

## Figures and Tables

**Table 1:** Comparison of group characteristics between patients who were treated with drug-coated balloon angioplasty (DCB) and with conventional angioplasty (POBA).

	DCB Group (*n* = 38)	POBA Group (*n* = 38)	*p*
age	71 (IQR 65, 78)	69 (IQR 64, 76)	0.557
male sex	21 (55%)	18 (47%)	0.647
antipletelet therapy	22 (58%)	26 (68%)	0.476
anticoagulation therapy	15 (39%)	12 (32%)	0.632
diabetes	25 (66%)	23 (61%)	0.812
ischemic heart disease	13 (34%)	13 (34%)	1.0
AVF	26 (68%)	28 (74%)	0.800
AVG	12 (32%)	10 (26%)	
median time since access creation (years)	1.56 (IQR 0.89, 2.91)	1.42 (IQR 0.92, 2.74)	0.934
previous PTA on vascular access	29 (76%)	21 (82%)	0.779
stenosis location			0.687
graft	2 (5%)	1 (3%)	
outflow vein	11 (29%)	8 (21%)	
juxta-anastomotic outflow vein	21 (55%)	26 (68%)	
venous anastomosis	4 (11%)	3 (8%)	
balloon diameter			0.786
5 mm	5 (13%)	5 (13%)	
6 mm	18 (47%)	21 (52%)	
7 mm	12 (32%)	10 (26%)	
8 mm	3 (8%)	2 (5%)	
degree of stenosis	67% ± 11%	69% ± 10%	0.360

DCB, drug-coated balloon; POBA, conventional angioplasty; AVF, arteriovenous fistula; AVG, arteriovenous graft; 95%CI, 95% confidence interval; IQR, interquartile range.

## Data Availability

Data from the study are available upon reasonable request from the corresponding author.
